# Associations between impulsivity and fecal microbiota in individuals abstaining from methamphetamine

**DOI:** 10.1111/cns.14580

**Published:** 2024-02-08

**Authors:** Wen Liu, Linzi Liu, Zijing Deng, Ruina Liu, Tao Ma, Yide Xin, Yu Xie, Yifang Zhou, Yanqing Tang

**Affiliations:** ^1^ Department of Psychiatry The First Hospital of China Medical University Shenyang Liaoning PR China; ^2^ Department of Psychiatry The First Affiliated Hospital of Xi'an Jiaotong University Xi'an Shanxi PR China; ^3^ Department of Psychiatry Shengjing Hospital of China Medical University Shenyang Liaoning PR China

**Keywords:** abstention, impulsivity, methamphetamine, microbiota, microbiota–gut–brain axis

## Abstract

**Introduction:**

Methamphetamine (MA) abuse is a major public problem, and impulsivity is both a prominent risk factor and a consequence of addiction. Hence, clarifying the biological mechanism of impulsivity may facilitate the understanding of addiction to MA. The microbiota–gut–brain axis was suggested to underlie a biological mechanism of impulsivity induced by MA.

**Methods:**

We therefore recruited 62 MA addicts and 50 healthy controls (HCs) to investigate the alterations in impulsivity and fecal microbiota and the associations between them in the MA group. Thereafter, 25 MA abusers who abstained from MA for less than 3 months were followed up for 2 months to investigate the relationship between impulsivity and microbiota as abstinence became longer. 16S rRNA sequencing was conducted for microbiota identification.

**Results:**

Elevated impulsivity and dysbiosis characterized by an increase in opportunistic pathogens and a decrease in probiotics were identified in MA abusers, and both the increased impulsivity and disrupted microbiota tended to recover after longer abstinence from MA. Impulsivity was related to microbiota, and the effect of MA abuse on impulsivity was mediated by microbiota.

**Conclusion:**

Our findings potentially highlighted the importance of abstention and implicated the significant role of the microbiota–gut–brain axis in the interrelationship between microbiota and behaviors, as well as the potential of microbiota as a target for intervention of impulsivity.

## Graphical abstract

(1) Disrupted intestinal microbiota in methamphetamine abusers was associated with impulsivity levels in both baseline and after 2 more months follow‐up. (2) The effect of MA abuse on impulsivity was mediated by intestinal microbiota.

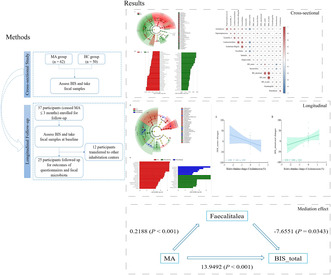

## 1 INTRODUCTION

The substantial use of methamphetamine (MA) is a major public health problem worldwide. More than 1.6 million adults used MA from 2015 to 2018 in the United States,^1^ and MA accounted for a high rate of 48% of abused drugs in Shanghai, China, from 2018 to 2019.^2^ MA abuse was linked to neurocognitive impairment, neuropsychiatric disorders, and higher hospital costs^3^, ^4^, ^5^, ^6^ and generated 11% of the accumulated amount of overdose deaths in the United States.^7^ Impulsivity has been suggested to be involved in the neurobiological mechanism of addiction,^8^ and impulsive individuals are more vulnerable to addiction relapse and continuity.^9^, ^10^ Therefore, clarifying the biological mechanism of impulsivity may facilitate the understanding of addiction in MA.

Impulsivity is a multidimensional construct characterized by disinhibition and an unplanned manner to satisfy an immediate desire,^11^ which is both a prominent risk factor and a consequence of addiction.^12^ Impulsivity endophenotypes were suggested to precede addiction behaviors^13^ and have been taken as a determinant of later addiction formation, where constant drug use may increase impulsivity and hence reinforce addiction behaviors in turn.^14^ Impulsivity was found to be positively related to use severity and negatively linked to the initial age of MA use.^15^, ^16^ Additionally, high‐impulsive MA abusers were younger, consumed larger amounts of MA, and were more likely to binge than low‐impulsive MA abusers.^17^ Furthermore, current MA abusers exhibited the highest impulsivity, while past MA abusers were between healthy controls and current MA abusers in impulsivity level,^18^ indicating that impulsivity may be reduced after abstaining from MA. Interestingly, impulsivity was lower in MA abusers with longer abstinence durations than in those with shorter durations,^19^, ^20^ but the underlying mechanism remains unclear. Houdek proposed that microbiota could affect behaviors including impulsivity,^21^ and the microbiome–gut–brain axis (MGBA) was suggested to be implicated in the reward process that was related to impulsivity.^22^ Therefore, we propose that MGBA may underlie a biological mechanism of impulsivity induced by MA.

The microbiome–gut–brain axis (MGBA) is a bidirectional communication system between the brain and the gut microbiota^23^ that maintains the internal homeostasis of the host by immune, neural, endocrine, and metabolic pathways.^24^, ^25^ Both compositional and structural alterations of gut microbiota were observed in MA abusers,^26^, ^27^ and the alterations were related to psychiatric symptoms and cognitive dysfunctions.^27^ Additionally, intestinal microbiota was also suggested to potentially differentiate MA abusers from healthy group.^28^ In animal studies, MA exposure was shown to induce gut microbiota dysbiosis, intestinal barrier impairment, neuroinflammation elevation, and behavioral abnormalities.^29^, ^30^ However, the neurotoxic effects induced by MA could be ameliorated by optimizing gut homeostasis.^30^ Moreover, microbiota disruption has been proposed as a causal effect of these alterations.^30^, ^31^ For example, the formation of MA addiction was inhibited through the depletion of gut microbiota with antibiotics, while the inhibition was reversed by microbiota transplantation.^32^ A similar conclusion was confirmed by Chen et al. in morphine addiction models, where the formation and treatment effect of addiction was through microbiota.^33^ In addition, an improvement in impulsivity induced by probiotics was also identified in a double‐blind randomized clinical study.^34^ Taken together, these results suggest the bidirectional pathway of MGBA and the important role of microbiota as a potential target for treatment. Furthermore, Forouzan et al. claimed that the disrupted gut microbiota was reversible after MA cessation in an animal study.^35^ However, there are still some questions to be answered, for instance, whether the altered microbiota induced by MA recovers in humans and what is the relationship between elevated impulsivity and disrupted gut microbiota?

Therefore, in the current study, we planned to answer these questions based on both cross‐sectional and longitudinal studies. We proposed that 1) impulsivity and microbiota were altered in MA abusers, and the increased impulsivity was associated with microbiota in MA abusers; 2) the disrupted microbiota might recover as abstinence duration becomes longer, and the changes in impulsivity were related to the changes in microbiota.

## 2 METHODS AND MATERIALS

### 2.1 Participants

Sixty‐two inpatients were recruited from the First Rehabilitation Center of Shenyang, and all participants met the diagnosis of methamphetamine use disorder (MUD). Exclusion criteria were as follows: mixed use of other addictive drugs such as opioids, cocaine, and marijuana; history of psychiatric diagnoses other than MUD; medical conditions that may alter the fecal microbiota, such as autoimmune diseases, metabolic diseases, diabetes, cirrhosis, fatty liver, hepatitis, inflammatory bowel disease, HIV, and syphilis; and taking antibiotics or probiotics within 1 month. Fifty healthy controls (HCs) matched based on sex and age were recruited from the community through advertisement. The exclusion criteria were the same as those for the MA group, and all HCs reported no history of illegal drug use. Twenty‐five MA abusers who abstained from MA for less than 3 months were then followed up for 2 months to investigate how impulsivity and fecal microbiota may be altered as abstinence became longer (see Supplementary Figure S1). For all participants, the Barratt Impulsiveness Scale‐11 (BIS‐11)^36^ was used to estimate impulsivity. Written informed consent was obtained from all participants. The Ethics Committee of the First Hospital of China Medical University approved the study (No. [2021]361).

### 2.2 Fecal samples, DNA extraction, and 16S rRNA sequencing

Fresh fecal samples from all participants were collected in sterile containers with fecal preservation agent (CW2654, CwBiotech, Beijing, China) and stored at −80°C until DNA extraction. We used MN NucleoSpin 96 Soikit for DNA extraction and applied the metabarcoding approach developed by Miya for analysis.^37^ The V3‐V4 region of the bacterial 16S rRNA gene was amplified by 2 rounds of PCR. The first round of PCR was as follows: 95°C for 5 min, followed by 25 cycles at 95°C for 30 s, 50°C for 30 s, 72°C for 40 s, and then 72°C for 7 min for final extension with primers 338F (5′‐ ACTCCTACGGGAGGCAGCA‐3′) and 806R (5′‐ GGACTACHVGGGTWTCTAAT‐3′), and the second round of PCR was under 98°C for 30 s, followed by 10 cycles at 98°C for 10 s, 65°C for 30 s, 72°C for 40 s, and a final extension at 70°C for 5 min. Finally, the amplicons were extracted from 1.8% agarose gels using a Monarch DNA extraction kit.

The raw reads were extended using FLASH (version 1.2.11)^38^ and filtered with Trimmomatic (version 0.33),^39^ and chimeras were removed with UCHIME (version 8.1).^40^ Then, we obtained clean tags. The clear reads were clustered into amplicon sequence variants (ASVs), which had finer resolution so that errors could be sufficiently controlled.^41^

### 2.3 Statistical analysis

Demographic and clinical analyses were performed using SPSS v26.0 (SPSS, Chicago, IL, USA). The Kolmogorov–Smirnov test was performed for normality test. For normally distributed data, analysis of variance (ANOVA) and *t* tests were applied for group comparisons across 3 groups or 2 groups, respectively, and Kruskal–Wallis and Mann–Whitney tests were performed for nonnormally distributed variables across 3 groups or 2 groups, respectively. The chi‐square test (*x* ^2^ test) was used for categorical variables. Body mass index (BMI) was calculated by weight (kg) divided by the square of height (m). Alpha diversity was computed based on ASV levels, including the ACE index, Chao 1 index, Simpson index, and Shannon index. Beta diversity was calculated using an unweighted Bray–Curtis distance matrix at the ASV level, which was then subjected to principal component analysis (PCA). Permutational multivariate analysis of variance (PERMANOVA) was used to quantitatively compare the Bray–Curtis distance across groups. The linear discriminant analysis (LDA) effect size (LEfSe) was applied to identify the abundant taxa with a threshold of LDA score >2. To identify the association between the abundance of fecal microbiota and impulsivity, Spearman's correlation was applied for correlation analysis, and the false discovery rate (FDR) was used for correction. The significance threshold was set as *p* < 0.05. Mediation analysis was performed to investigate the relationship between MA abuse, fecal microbiota, and impulsivity, in which MA abuse was taken as an independent variable, fecal microbiota as a mediator, and impulsivity as a dependent variable. We applied nonparametric bootstrap analysis,^42^ where the mediation was significant if 0 was not included in the confidence intervals (CIs) with 95% bias correction.

## 3 RESULTS

### 3.1 Demographic characteristics

At baseline, a total of 112 participants (MA group: 62, HC group: 50) were included and their impulsivity was estimated, and no significant differences were observed between groups in age (U = −1.474, *p* = 0.141), sex (*x* ^2^ = 0.723, *p* = 0.395), smoking within 1 month (*x* ^2^ = 1.139, *p* = 0.286), and BMI (U = 1.265, *p* = 0.208) (Table 1). Twenty‐five participants who abstained from MA within 3 months were then followed up for 2 months, and HCs were significantly older than participants in the MA_baseline group and the MA_follow‐up group (H = 6.699, *p* = 0.035). Significant difference across MA_baseline, MA_follow‐up, and HC groups was identified in smoking within 1 month (*x* ^2^ = 11.552, *p* = 0.003). No significant differences were discovered in sex (*x* ^2^ = 0.164, *p* = 0.921) or BMI (H = 3.032, *p* = 0.053) across the three groups (Table 2).

**TABLE 1:** Demographic and clinical characteristics in MA and HCs.

	MA (*n* = 62)	HC (*n* = 50)	U*/t*/*χ* ^2^	*p*
Demographic characteristics				
Sex (female: male)	20:42	20:30	0.723	0.395
Age (years)	41.00 (34.00, 50.00)	43.50 (35.00, 53.00)	−1.474	0.141
BMI (kg/m^2^)	25.35 ± 3.69	24.54 ± 2.96	1.265	0.208
Smoking within 1 month (yes: no)	15:47	8:42	1.139	0.286
Clinical features				
BIS_total	43.36 ± 13.42	31.08 ± 10.08	5.361	<0.001
BIS_motor	41.25 (27.50, 55.00)	30.00 (15.00, 40.00)	4.128	<0.001
BIS_attentional	42.50 (30.00, 52.50)	33.75 (27.50, 42.50)	2.279	0.023
BIS_no plan	41.25 (22.50, 55.00)	31.25 (22.50, 40.00)	2.534	0.011

Abbreviations: HC, healthy controls; MA, participants who are absent from methamphetamine. *Note*: Normally distributed data were compared with *t* test and exhibited in mean ± SD, while the nonnormally distributed one was compared with Mann–Whitney and presented in median (lower quartile and upper quartile). Threshold of significant difference was set at *p* < 0.05.

**TABLE 2:** Demographic and clinical characteristics across MA_baseline, MA_follow‐up, and HC groups.

	MA_baseline (*n* = 25)	MA_follow‐up (*n* = 25)	HC (*n* = 50)	H/F/*χ* ^2^	*p*
Demographic characteristics					
Sex (female: male)	11:14	11:14	20:30	0.164	0.921
Age (years)	39.00 (27.50, 49.00)	39.00 (27.50, 49.00)	43.50 (34.75, 53.00)	6.699	0.035
BMI (kg/m^2^)	26.03 ± 3.48	26.20 ± 3.39	24.54 ± 2.96	3.032	0.053
Smoking within 1 month (yes: no)	9:16	0:25	8:42	11.552	0.003
Clinical features					
BIS_total	46.77 ± 10.41	39.17 ± 12.94	31.08 ± 10.08	17.780	< 0.001
BIS_motor	40.00 (28.75, 52.50)	27.50 (16.25, 72.50)	30.00 (14.98, 40.00)	7.068	0.029
BIS_attentional	50.70 ± 14.97	39.50 ± 23.11	34.60 ± 11.58	8.470	< 0.001
BIS_no plan	47.50 (38.75, 61.25)	27.50 (20.00, 51.25)	31.25 (22.50, 40.63)	14.751	< 0.001
Withdraw duration (days)	44.28 ± 28.79	104.72 ± 45.60	NA	5.604	< 0.001

Abbreviations: HC, healthy controls; MA_baseline group, participants who ceased methamphetamine within 3 months; MA_follow‐up group, participants followed up for 2 months from baseline; NA, not applicable. *Note*: Normally distributed data were compared with ANOVA or t tests and are presented as the mean ± SD, while nonnormally distributed data were compared with the Kruskal–Wallis test and are presented as the median (lower quartile and upper quartile). The Bonferroni correction was applied for post hoc analysis, and the threshold of significant difference was set at *p* < 0.05.

### 3.2 Clinical characteristics

**FIGURE 1:**
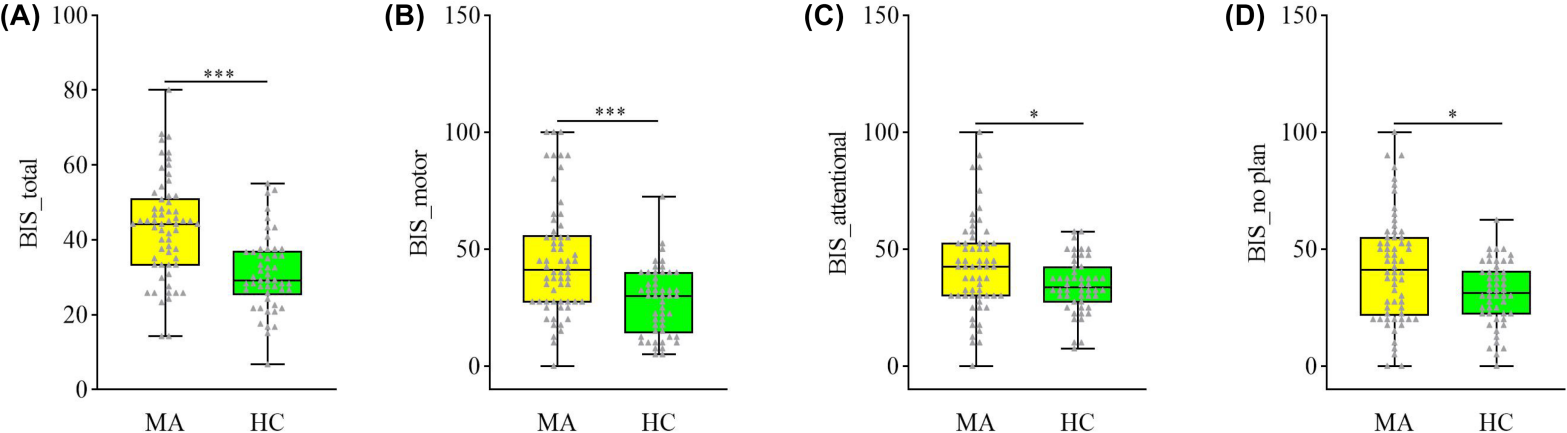
Impulsivity variation between the MA and HC groups. Significant differences were observed between the MA group and HC group in BIS_total, BIS_motor, BIS_attentional, and BIS_no plan. In Figure (A), BIS_total was obtained through the *t* test, while the results from (B–D) were obtained through the Mann–Whitney test. The threshold of significant difference was set at *p* < 0.05. HC, healthy controls; MA, participants who are absent from methamphetamine. ***p < 0.001, *p < 0.05.

**FIGURE 2:**
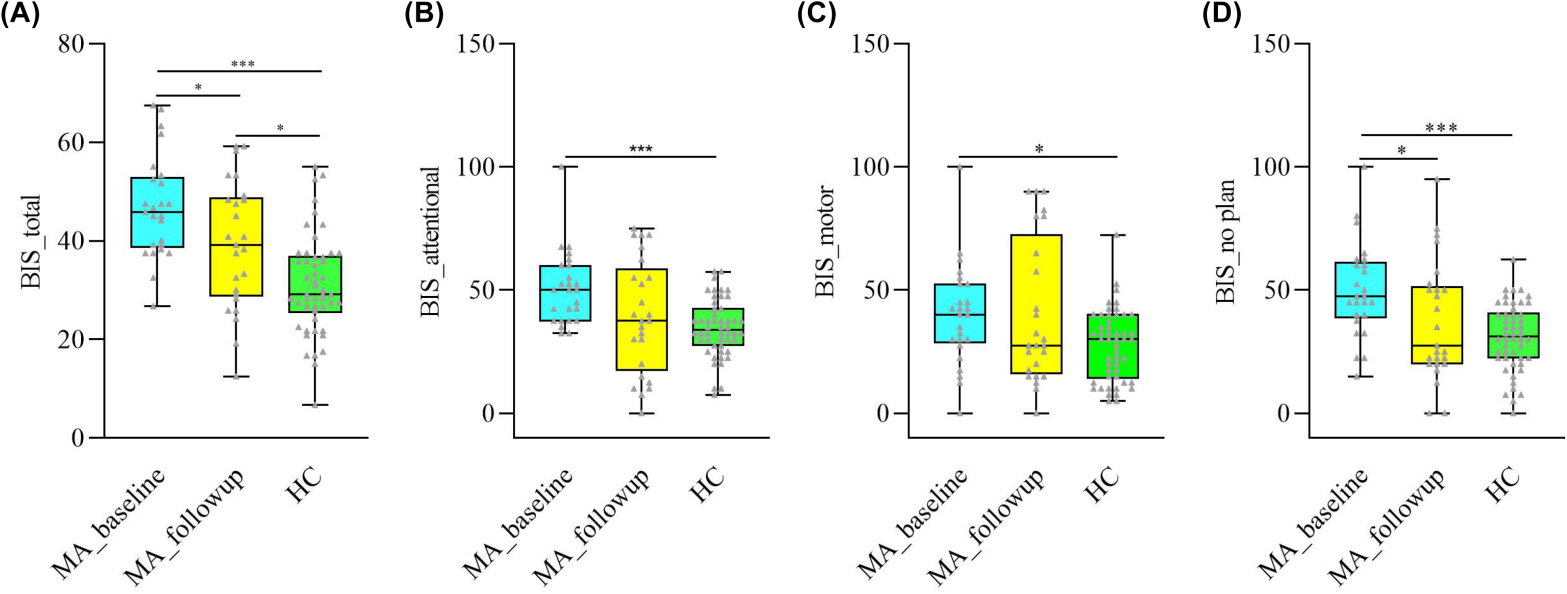
Impulsivity variation across the MA_baseline, MA_follow‐up, and HC groups. Significant differences were observed across the MA_baseline, MA_follow‐up, and HC groups in BIS_total, BIS_motor, BIS_attentional, and BIS_no plan. In Figure (A and B) BIS_total and BIS_attentional were identified using ANOVA, while the results from (B–D) were obtained through the Kruskal–Wallis test. The Bonferroni correction was applied for post hoc analysis, and the threshold of significant difference was set at *p* < 0.05. MA_baseline group: participants who ceased methamphetamine within 3 months; MA_follow‐up group: participants followed up for 2 months from baseline; HC: healthy controls. ***p < 0.001, *p < 0.05.

For trait impulsivity, significantly higher scores were identified in the MA group relative to the HC group, namely BIS_total (*t* = 5.361, *p* < 0.001), BIS_motor (U = 4.128, *p* < 0.001), BIS_attentional (U = 2.279, *p* = 0.023), and BIS_no plan (U = 2.534, *p* = 0.011) (Table 1 and Figure 1). For 25 MA participants who were involved in the follow‐up, significant differences were detected across the MA_baseline, MA_follow‐up, and HC groups in BIS_total (F = 17.780, *p* < 0.001), BIS_motor (H = 7.068, *p* = 0.029), BIS_attentional (F = 8.470, *p* < 0.001), and BIS_no plan (H = 14.751, *p* < 0.001). The score in the MA_baseline group was significantly greater than in the HC group in BIS_total (*p* < 0.001), BIS_attentional (*p* < 0.001), BIS_motor (*p* = 0.029), and BIS_no plan (*p* < 0.001). After a 2‐month additional abstinence of MA, only the BIS_total score (*p* = 0.010) was still significantly higher in the MA_follow‐up group relative to the HC group, while no significant differences were identified between the 2 groups in BIS_attentional (*p* = 0.641), BIS_motor (*p* = 0.452), and BIS_no plan (*p* = 0.966). Additionally, a significant decrease was observed in BIS_total (*p* = 0.047) and BIS_no plan (*p* = 0.042) scores after a 2‐month follow‐up in the MA_follow‐up group compared with the MA_baseline group (Table 2, Figure 2).

### 3.3 Structural alterations of fecal microbiota after ceasing MA

**FIGURE 3:**
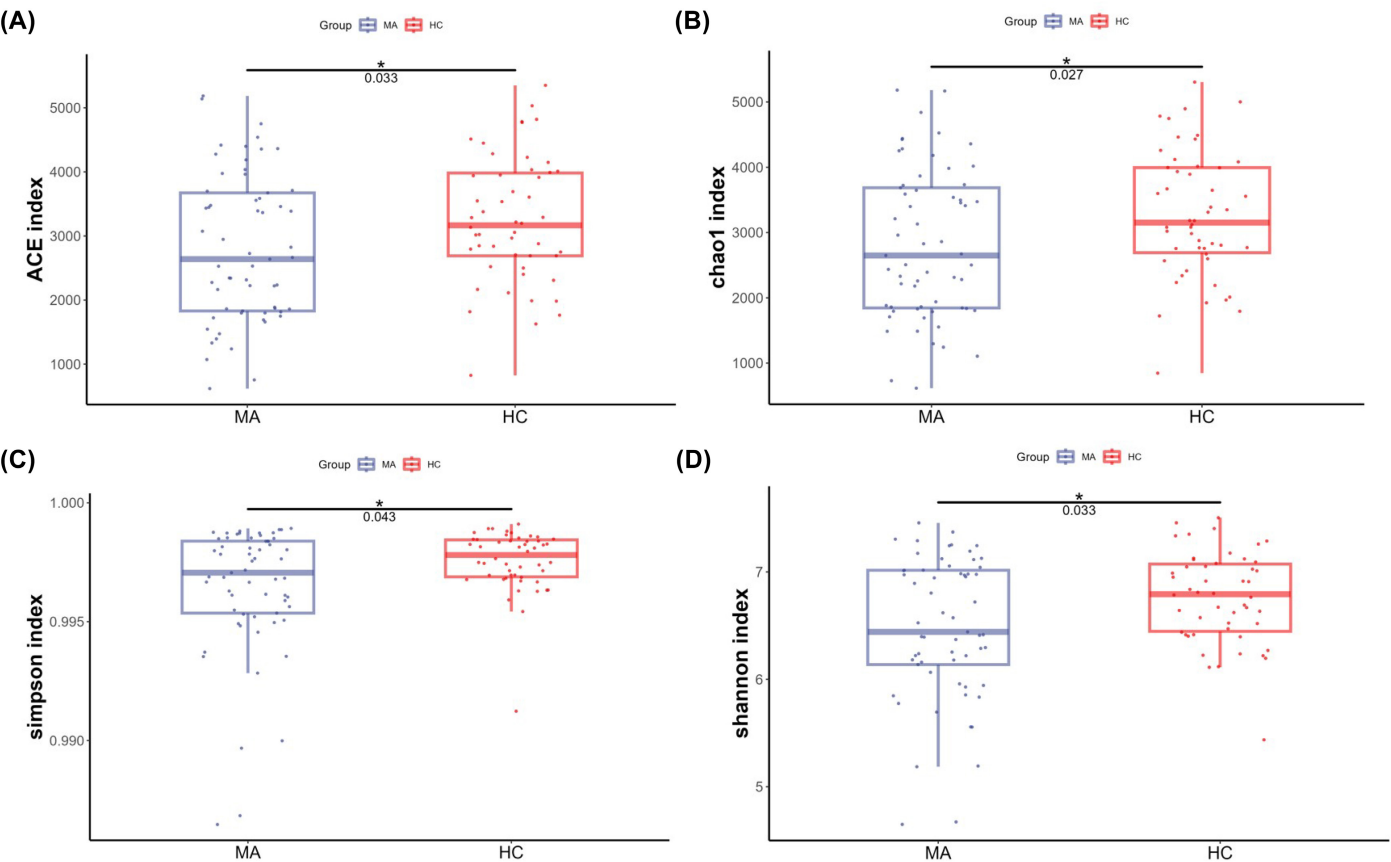
Alpha diversity between the MA and HC groups. There was a significant reduction in the ACE, Chao 1, Simpson, and Shannon indices in the MA group relative to the HC group. The threshold of significant difference was set at *p* < 0.05. MA: participants who are absent from methamphetamine, HC: healthy controls. *p < 0.05.

**FIGURE 4:**
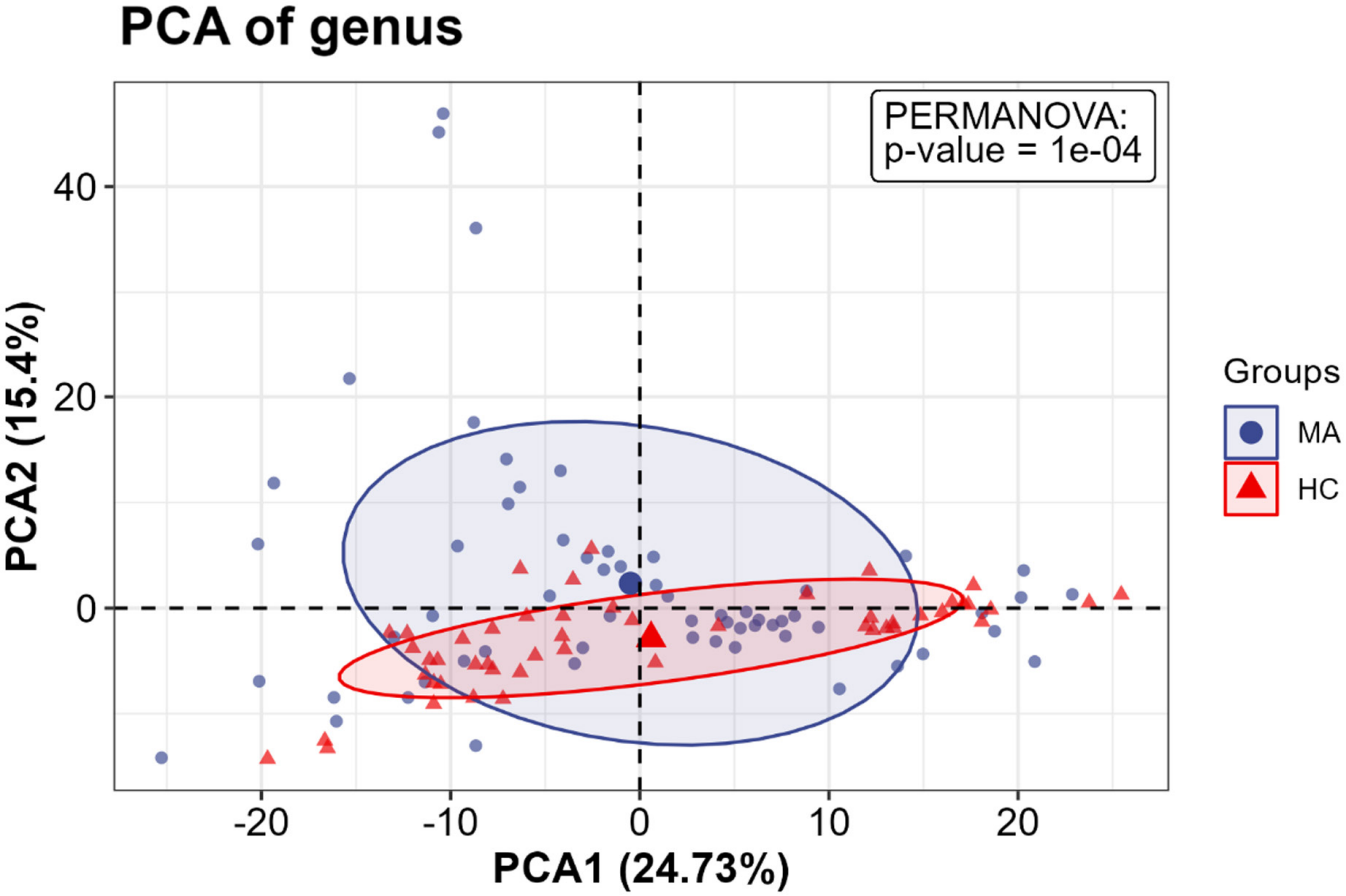
Beta diversity between the MA and HC groups. Significant separation at the genus level was identified using an unweighted Bray–Curtis matrix. PERMANOVA was applied to quantify the difference. The percentage represents the proportion of variance that can be explained by the corresponding axes. The threshold of significant difference was set at *p* < 0.05. MA: participants who are absent from methamphetamine, HC: healthy controls.

**FIGURE 5:**
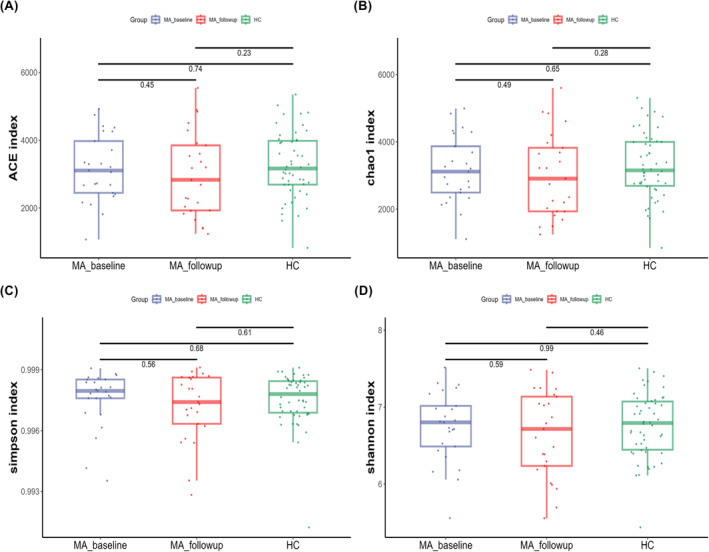
Alpha diversity across the MA_baseline, MA_follow‐up, and HC groups. No significant differences were observed in the ACE, Chao 1, Simpson, and Shannon indices across the 3 groups. The threshold of significant difference was set at *p* < 0.05. MA_baseline group: participants who ceased methamphetamine within 3 months; MA_follow‐up group: participants followed up for 2 months from baseline; HC: healthy controls.

**FIGURE 6:**
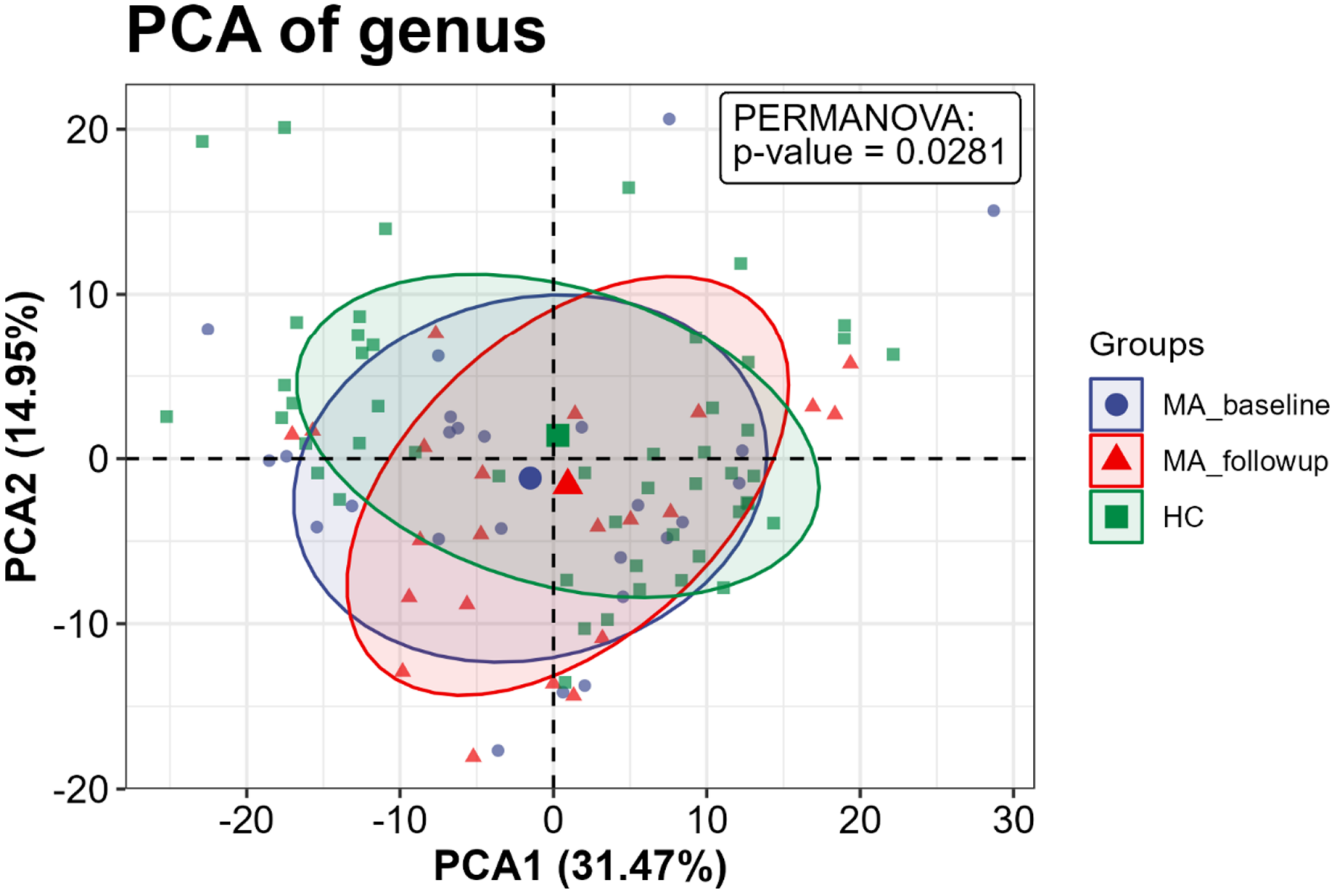
Beta diversity between the MA_baseline, MA_follow‐up, and HC groups. Significant separation at the genus level was identified using an unweighted Bray–Curtis matrix. PERMANOVA was applied to quantify the difference. The percentage represents the proportion of variance that can be explained by the corresponding axes. The threshold of significant difference was set at *p* < 0.05. MA_baseline group: participants who ceased methamphetamine within 3 months; MA_follow‐up group: participants followed up for 2 months from baseline; HC: healthy controls.

To investigate whether the fecal microbiota structure was altered in individuals who were abstinent from MA, alpha and beta diversity were compared at the ASV level between the MA and HC groups. The richness and diversity in the MA group were significantly lower than those in the HC group, as identified with the ACE (W = 1886, *p* = 0.033), Chao 1 (W = 1899, *p* = 0.027), Simpson (W = 1867, *p* = 0.043), and Shannon indices (W = 1885, *p* = 0.033) (Figure 3). A PCA plot based on unweighted Bray–Curtis at the genus level was applied to characterize the global differences in fecal microbiota between the MA group and HC group, and PERMANOVA was applied to quantify the difference. Figure 4 shows a significant separation between the MA and HC groups at the genus (*p* < 0.001) level. To identify whether the structure of the fecal microbiota was altered after an additional 2‐month abstinence, alpha and beta diversity were examined across the MA_baseline, MA_follow‐up, and HC groups. No significant differences were observed in the ACE (H = 1.470, *p* = 0.479), Chao 1 (H = 1.300, *p* = 0.522), Simpson (H = 0.497, *p* = 0.780), and Shannon (H = 0.568, *p* = 0.753) indices across the 3 groups (Figure 5). Meanwhile, a significant separation of the MA_baseline, MA_follow‐up, and HC groups (*p* = 0.0281) was identified at the genus level based on unweighted Bray–Curtis (Figure 6).

### 3.4 Compositional alterations of fecal microbiota after ceasing MA

#### 3.4.1 Different abundance between MA and HC

**FIGURE 7:**
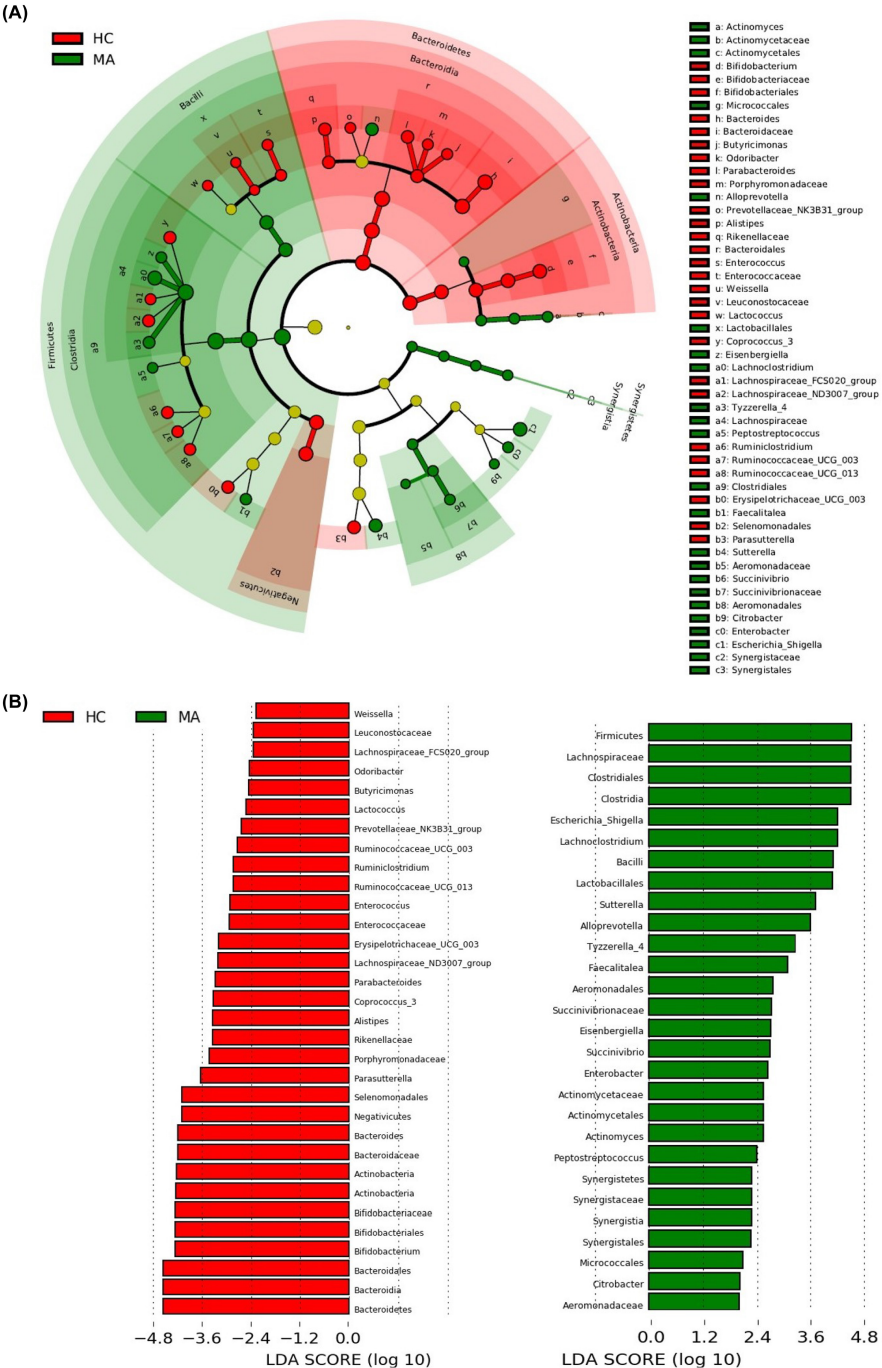
Taxonomic identification of fecal microbiota with different abundances between the MA and HC groups. (A) Significant differences in bacterial abundance were identified in various taxa using LEfSe. The inner to the outer circle represents phylum, class, order, family, and genus accordingly. Taxa with significantly different abundances are presented in different colors according to different groups. Taxa with no significant difference in abundance are shown in the yellow circle. (B) Linear discriminant analysis (LDA) exhibited the effect size of various taxa with a significant difference in abundance (LDA score >2, *p* < 0.05).

The fecal bacterial composition was altered significantly even after the cessation of MA abuse. LEfSe identified different taxa with significantly different abundances between the MA and HC groups (LDA score >2) (Figure 7). In particular, we observed significantly greater abundance in MA than HC in *Firmicutes* and *Synergistetes* at the phylum level; Bacilli, *Synergistia*, and Clostridia at the class level; *Actinomycetales*, *Micrococcales*, *Lactobacillales*, *Clostridiales*, *Aeromonadales*, and *Synergistales* at the order level; *Actinomycetaceae*, *Lachnospiraceae*, *Aeromonadaceae*, *Succinivibrionaceae*, and *Synergistaceae* at the family level; and *Escherichia_Shigella*, *Lachnoclostridium*, *Sutterella*, *Alloprevotella*, *Tyzzerella_4*, *Faecalitalea*, *Eisenbergiella*, *Succinivibrio*, *Enterobacter*, *Actinomyces*, *Peptostreptococcus*, and *Citrobacter* at the genus level. In contrast, significantly higher abundance in the HC group was observed relative to the MA group in the phyla *Actinobacteria* and *Bacteroidetes*; the classes *Actinobacteria*, *Bacteroidia*, and *Negativicutes*; the orders *Bifidobacteriales*, *Bacteroidales*, and *Selenomonadales*; the families *Bifidobacteriaceae*, *Bacteroidaceae*, *Porphyromonadaceae*, *Rikenellaceae*, *Enterococcaceae*, and *Leuconostocaceae*; the genera *Bifidobacterium*, *Bacteroides*, *Butyricimonas*, *Odoribacter*, *Parabacteroides*, *Prevotellaceae_NK3B31_group*, *Alistipes*, *Enterococcus*, *Weissella*, *Lactococcus*, *Coprococcus_3*, *Lachnospiraceae_FCS020_group*, *Lachnospiraceae_ND3007_group*, *Ruminiclostridium*, *Ruminococcaceae_UCG_003*, *Ruminococcaceae_UCG_013*, *Erysipelotrichaceae_UCG_003*, and *Parasutterella*. To quantify the group difference at the genus level, we further compared the relative abundance between the MA and HC groups using the Wilcoxon test, and FDR was applied for correction. The relative abundances of *Alloprevotella* (q = 0.037), *Enterobacter* (q = 0.009), *Faecalitalea* (q < 0.001), *Lachnoclostridium* (q = 0.036), and *Escherichia‐Shigella* (q = 0.045) were significantly enriched in the MA group compared with the HC group. Meanwhile, MA exhibited significantly reduced relative abundance in *Bacteroides* (q = 0.025), *Alistipes* (q = 0.036), *Parasutterella* (q = 0.011), *Erysipelotrichaceae_UCG‐003* (q = 0.025), *Bifidobacterium* (q = 0.003), *Lachnospiraceae_ND3007_group* (q < 0.001), and *Coprococcus_3* (q = 0.003) (Supplementary Figure 2).

#### 3.4.2 Different abundances across MA_baseline, MA_follow‐up, and HC groups

**FIGURE 8:**
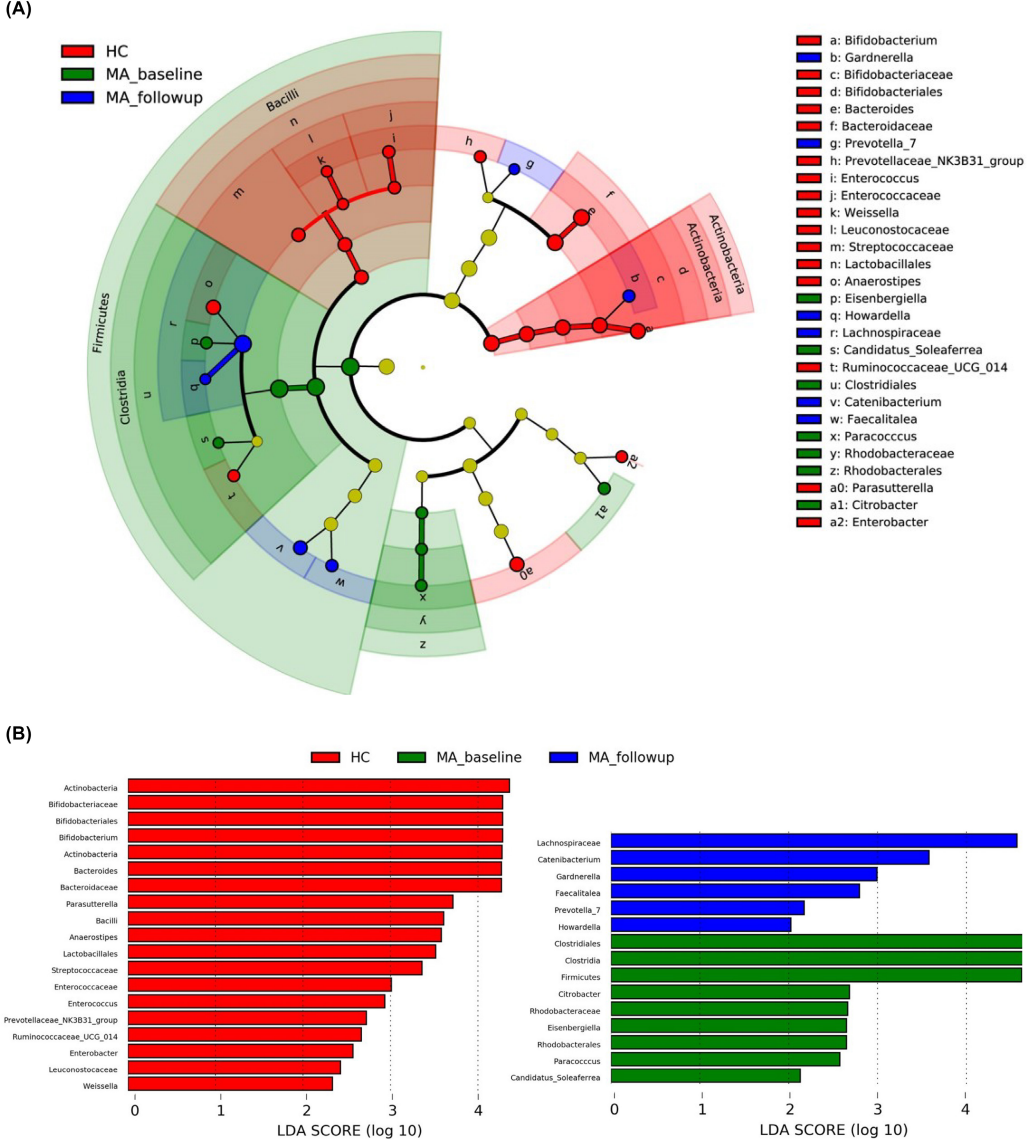
Taxonomic identification of fecal microbiota with different abundances across the MA_baseline, MA_follow‐up, and HC groups. (A) Significant differences in bacterial abundance were identified in various taxa using LEfSe. The inner to outer circles represent the phylum, class, order, family, and genus accordingly. Taxa with significantly different abundances are presented in different colors according to different groups. Taxa with no significant difference in abundance are shown in the yellow circle. (B) Linear discriminant analysis (LDA) exhibited the effect size of various taxa with a significant difference in abundance (LDA score >2, *p* < 0.05).

To investigate whether the composition of the fecal microbiota was altered after a 2‐month follow‐up, LEfSe was performed to identify the taxonomic variations across the MA_baseline, MA_follow‐up, and HC groups, with an LDA score >2 indicating a significant difference in abundance (Figure 8). Significantly enriched abundance was identified in the MA_baseline group in the phylum *Firmicutes*; the class *Clostridia*; the order *Clostridiales* and *Rhodobacterales*; the family *Rhodobacteraceae*; and the genera *Eisenbergiella*, *Candidatus_Soleaferrea*, *Paracoccus*, and *Citrobacter*. Meanwhile, the abundance was significantly greater in the MA_follow‐up group in *Lachnospiraceae* at the family level and in *Gardnerella*, *Prevotella_7*, *Howardella*, *Catenibacterium*, and *Faecalitalea* at the genus level. For the HC group, significantly elevated abundance was observed in the phylum *Actinobacteria*; the class *Bacilli*; the orders *Bifidobacteriales* and *Lactobacillales*; the families *Bifidobacteriaceae*, *Bacteroidaceae*, *Enterococcaceae*, *Leuconostocaceae*, and *Streptococcaceae*; and the genera *Bifidobacterium*, *Bacteroides*, *Prevotellaceae_NK3B31_group*, *Enterococcus*, *Weissella*, *Anaerostipes*, *Ruminococcaceae_UCG_014*, *Parasutterella*, and *Enterobacter*. Likewise, relative abundance across 3 groups at the genus level was calculated using the Kruskal–Wallis test, and FDR was applied for correction. Significant differences were identified across the MA_baseline, MA_follow‐up, and HC groups in *Arthrobacter* (q < 0.001), *Weissella* (q < 0.001), *Enterobacter* (q = 0.002), *Bifidobacterium* (q = 0.004), and *Klebsiella* (q = 0.019) (Supplementary Figure 3). Furthermore, relative abundance was significantly reduced in *Enterococcus* (*p* = 0.040) and increased in *Acidaminococcus* (*p* = 0.049) in the MA_follow‐up group relative to the MA_baseline group.

### 3.5 Associations between fecal microbiota and impulsivity in the MA group

**FIGURE 9:**
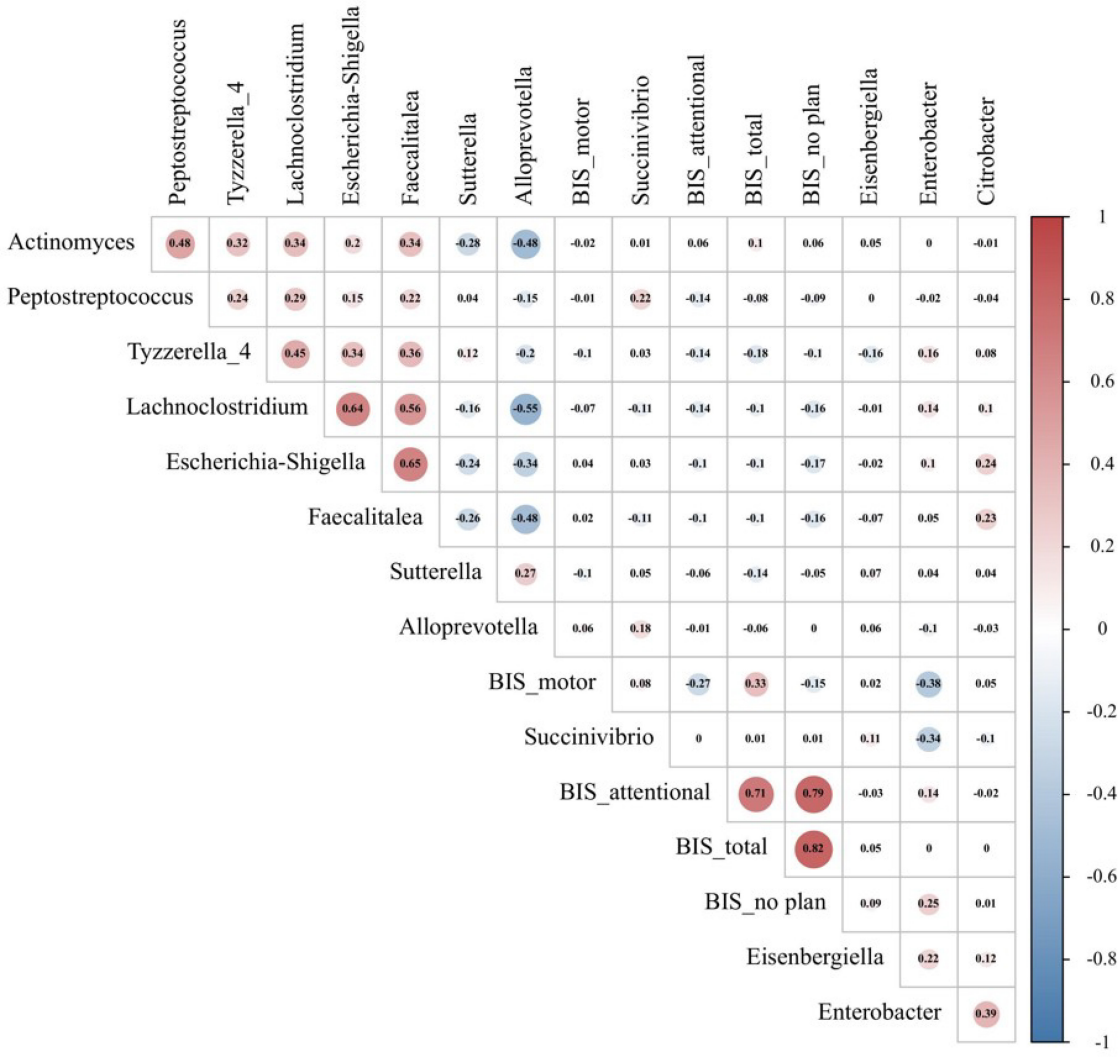
Association between impulsivity and relative abundance of fecal microbiota. Red represents a positive association, and blue indicates a negative association. The correlation coefficient is presented in the plot, the size of which is reflected by the size of the circle.

**FIGURE 10:**
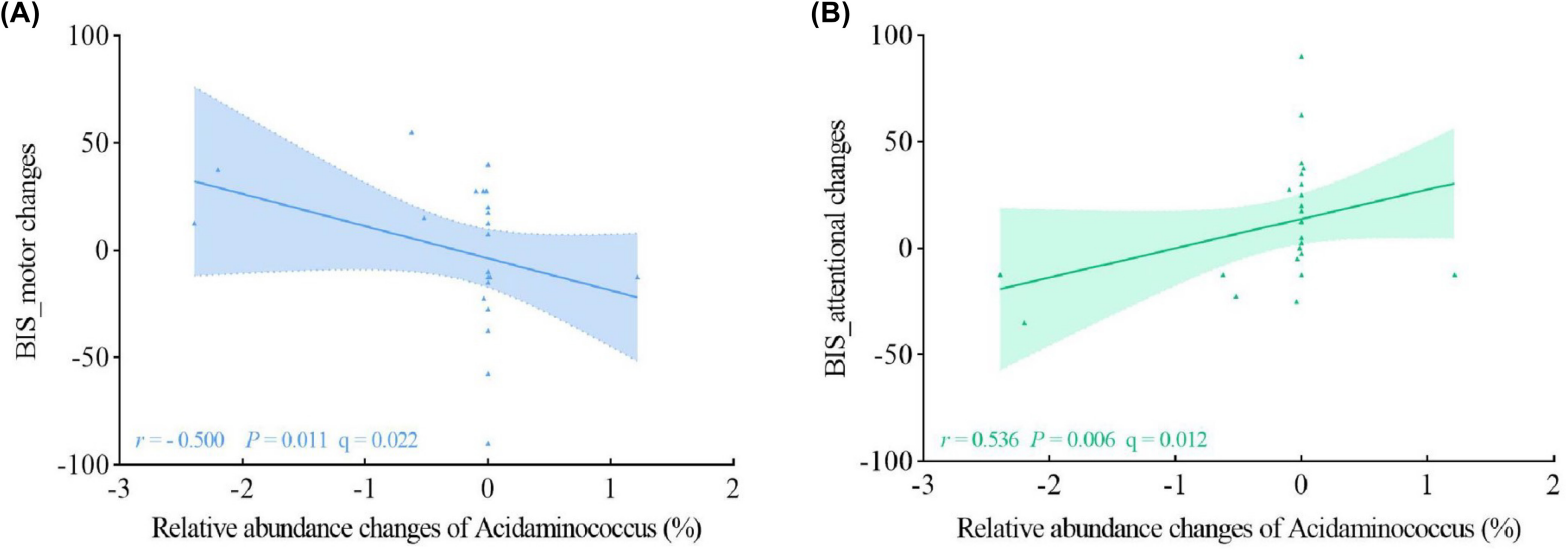
Associations between impulsivity changes and relative abundance changes of fecal microbiota. (A) Relative abundance chances of *Acidaminococcus* negatively correlated with changes of BIS_motor. (B) Relative abundance chances of *Acidaminococcus* positively correlated with BIS_attentional.

We investigated the relationship between fecal microbiota and impulsivity in MA at both the cross‐sectional level and the longitudinal level using Spearman's correlation with FDR for correction. In the cross‐sectional study, we investigated the associations between impulsivity and the relative abundance of fecal microbiota with LDA score >2 at the genus level in the MA group. Therefore, we observed a negative association between BIS_motor and the relative abundance of *Enterobacter* (*r* = −0.381, *p* = 0.002) (Figure 9), and the correlation survived after FDR correction (*r* = −0.381, q = 0.024). To further dynamically investigate the relationship between impulsivity and fecal microbiota, associations between changes in impulsivity and relative abundance of fecal microbiota with significant differences between the MA_baseline group and the MA_follow‐up group at the genus level were estimated. Thus, we observed that changes in the relative abundance of *Acidaminococcus* were negatively correlated with changes in BIS_motor (*r* = − 0.500, *p* = 0.011) and positively related to BIS_attentional (*r* = 0.536, *p* = 0.006). The associations both survived the FDR correction (Figure 10).

### 3.6 Mediation effects between MA abuse, impulsivity, and fecal microbiota

**FIGURE 11:**
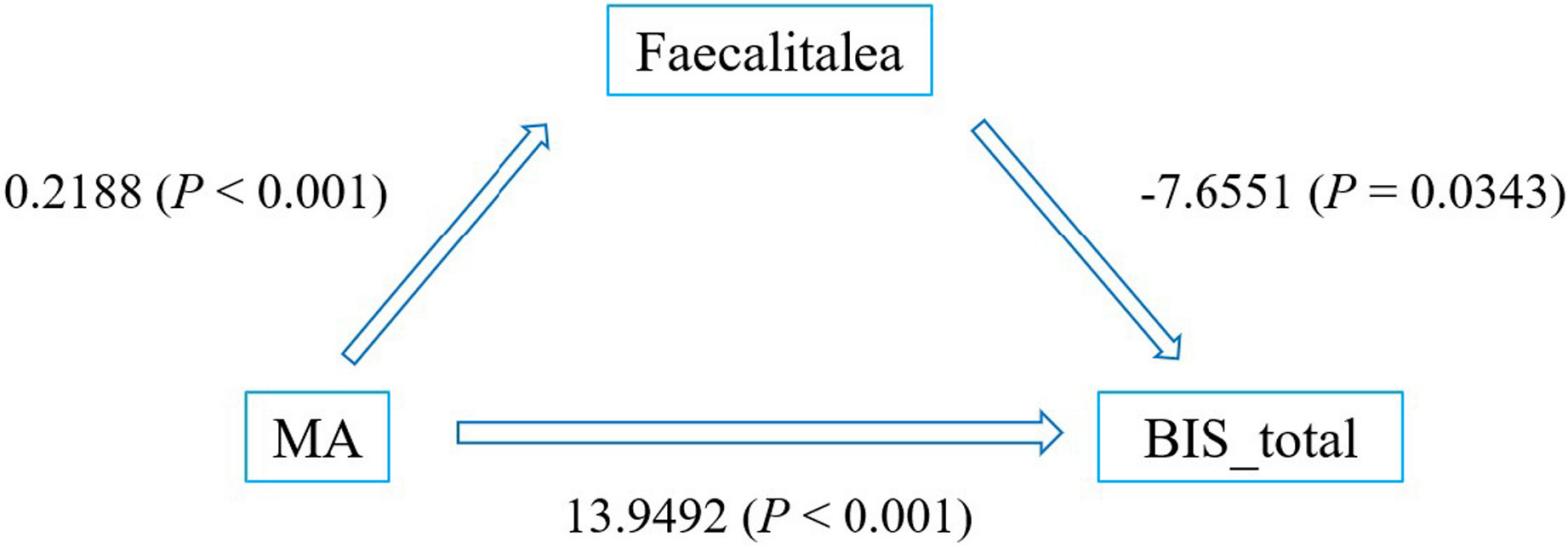
The relative abundance of Faecalitalea mediated the association between MA abuse and BIS_total scores. MA usage negatively predicted BIS_total through Faecalitalea with an indirect effect and positively predicted BIS_total with a direct effect.

To explore the relationship between MA abuse, impulsivity, and fecal microbiota (with LDA score >2 at the genus level), mediation analysis was applied with 5000 bootstrapped samples. Therefore, we identified an indirect mediation effect (IE_Faecalitalea_ = −1.6751, LL = −3.2710, UL = −0.1707) from MA abuse to BIS_total, with a mediator of *Faecalitalea*, indicating that MA abusers tend to have a greater abundance of *Faecalitalea* thus leading to a lower level of impulsivity. The ratio of the indirect effect to the total effect was 13.65%. The paths from MA abuse to relative abundance of *Faecalitalea* (β = 0.2188, SE = 0.0602, *p* < 0.001), from relative abundance of *Faecalitalea* to BIS_total (β = −7.6551, SE = 3.5708, *p* = 0.0343), and from MA abuse to BIS_total (β = 13.9492, SE = 2.3847, *p* < 0.001) were all significant (Figure 11).

## 4 DISCUSSION

To the best of our knowledge, this is the first study to dynamically investigate the relationship between impulsivity and fecal microbiota in MA abusers. We observed distinctive structural and compositional fecal microbiota alterations in participants who ceased MA abuse. Additionally, associations between specific fecal microbiota and impulsivity, as well as the mediating role of microbiota in the effect of MA abuse on impulsivity, were identified in the study. Therefore, this study furthers our understanding of how MA affects impulsivity and provides new insights into potential intervention targets for the impulsivity of MA abusers in the future.

Diminished diversity and richness of fecal microbiota and imbalanced bacterial structure were observed in both cross‐sectional and longitudinal studies in the MA group, indicating a distinguishable variation in fecal microbiota after MA abuse. Similar to our finding, reduced diversity has also been identified in both human and animal studies, but different richness alterations in fecal microbiota were reported previously.^27^, ^43^ Compositional abnormalities were also detected in the current study. For example, *Enterobacter* and *Escherichia‐Shigella*, as members of the family *Enterobacteriaceae*, are opportunistic pathogens in humans and were both found to be predominant in the MA group relative to HCs. Both *Enterobacter* and *Escherichia‐Shigella* were common in immunocompromised patients such as newborn babies, patients with malignant tumors, mellitus diabetes, and critical illness.^44^, ^45^, ^46^, ^47^, ^48^ In addition, *Enterobacter* infections have also been frequently reported in heroin addicts,^49^, ^50^ which confirmed the immune system dysfunction of drug addicts.^51^ The elevated abundances of *Alloprevotella* and *Lachnoclostridium* identified in the MA group were both related to intestinal epithelial barrier damage^52^ and thus affected the behaviors and neuropsychiatric pathogenesis of the brain through MGBA.^53^ For example, *Alloprevotella* was considered a causative factor for the increase in trimethylamine N‐oxide,^54^ which has been shown to induce cognitive dysfunction by increasing senescent cells in the hippocampus and impairing synaptic structure and function.^55^ Furthermore, the abundance of *Escherichia‐Shigella* was positively associated with a peripheral inflammatory level in cognitively impaired patients,^56^ implying the immune pathway of MGBA communication.

Abundance reduction of probiotics such as *Bacteroides* and *Bifidobacterium* was another characteristic of MA abusers. *Bacteroides* is a beneficial bacterium that produces γ‐aminobutyric acid (GABA),^57^ modulates immunoreactions,^58^ metabolizes saccharides, and furnishes the host and other symbiotic microbiota with nutrition.^59^ Eliminated abundance of *Bacteroides* was also reported previously in MA abusers,^60^ while the abundance of *Bacteroides* was greater at the MA extinction stage relative to the acquisition stage in an animal study,^43^ hinting at a variation in the bacterial abundance at a different stage of MA abuse. *Bifidobacterium* is an anti‐inflammatory genus that is beneficial to intestinal barrier function and therefore protects epithelial cells from pathogenic invasion.^61^, ^62^ Meanwhile, *Bacteroides* modulates the host's immunologic development and differentiation and thus enhances the intestinal mucus barrier.^63^, ^64^ Hence, we speculate that the characteristic dysbiosis of a decrease in probiotics and an increase in opportunistic pathogens in the MA group may affect behaviors through disruption of the intestinal barrier and microbiota translocation.^65^ To further investigate the dynamic alterations in fecal microbiota within the MA group, a direct comparison between the MA_baseline group and the MA_follow‐up group was conducted, and a significant reduction in *Enterococcus* and an increase in *Acidaminococcus* were identified after 2 more months of MA ablation. *Enterococcus*, a common pathogen of infective endocarditis in drug abusers,^66^ is associated with dysfunction of the intestinal barrier.^67^ Meanwhile, *Acidaminococcus* produces butyric acids,^68^ which reduce intestinal permeability, enhance microbiota diversity, and enrich the abundance of probiotics.^69^ This increase in probiotics and decrease in pathogens suggests a reversible fecal microbiota alteration after longer MA abstinence, but whether it can recover to a normal range and how long it takes requires further studies with longer follow‐up.

MA participants had greater impulsivity than HCs in the cross‐sectional study, which was in alignment with previous findings.^16^ Interestingly, we observed a mediating effect of microbiota on the effect of MA abuse on impulsivity in the current study, suggesting an unneglectable role of MGBA. In the longitudinal observation, higher impulsivity was identified in MA abusers with shorter abstinence duration (<3 months), but the increased impulsivity recovered to a normal level except for the BIS_total scores after 2 more months of MA abstinence. A decline in impulsivity in MA abusers with longer abstinence duration was also demonstrated in a previous cross‐sectional study.^19^ However, Jones et al. reported a significant increase in impulsivity after 6 more days of abstinence in MA abusers,^70^ which may be due to the short abstinence of MA (mean duration of 0.87 days) at baseline and insufficient follow‐up duration. Furthermore, to explore the relationship between fecal microbiota and impulsivity, correlation analysis was conducted in both the cross‐sectional and longitudinal studies. An interesting phenomenon was that the increased abundance of *Acidaminococcus* was negatively correlated with the reduction in motor impulsivity but positively related to the decline in attentional impulsivity, exhibiting a different variation rate of motor impulsivity and attentional impulsivity as MA abstention became longer. Attentional impulsivity measures the ability to concentrate on the task at hand, while motor impulsivity refers to acting on the spur of the moment.^71^ Although both motor impulsivity and attentional impulsivity were positively related to disinhibition,^72^ the underlying mechanisms can be different. Increased attentional impulsivity mediated the effect of MA on deficits of time perception (overestimation of time intervals), while no such effect was identified in motor impulsivity.^73^ Moreover, treatment motivation was predicted by motor impulsivity, while craving severity was predicted by attentional impulsivity during drug detoxification,^74^ which exhibited various underlying impacts of multiple dimensions of impulsivity on MA abusers. Meanwhile, we identified a negative correlation between motor impulsivity and *Enterobacter* in the cross‐sectional observation. Higher motor impulsivity was associated with thinner prefrontal gray matter and greater volume of the nucleus accumbens and the amygdala, as well as greater functional connectivity within the basal ganglia–thalamus–cortical network.^75^, ^76^, ^77^ Additionally, impulsivity was observed to be associated with fecal microbiota as well as alterations in striatal dopamine receptors (D1R and D2R),^78^ and the high impulsivity level induced by MA may be due to inner activities in the mesocorticolimbic system influenced by D2R signaling in the striatum.^79^ Microbiota‐derived signals to neurons were suggested to affect the brain and behaviors.^80^ For example, *Enterobacter* and its derived metabolites are involved in the modulation of brain networks, including the striatum, amygdala, hippocampus thalamus, etc.,^81^ hinting at a potential pathway from the microbiota to the brain through modulation of molecular signals and brain networks.

There are some limitations that should be considered in the current study. First, the sample size was relatively small, especially in the longitudinal part, which made it difficult to generalize the results. Second, it was difficult to control the diet between the MA and HC groups, as the MA abusers were all in the inhabitation center, but this problem was avoided in the longitudinal part. Third, the follow‐up was only 2 months, so larger sample size studies with longer follow‐up are expected to identify whether MA‐induced dysbiosis could recover to the normal range. Fourth, although the MA group participants have ceased smoking before feces collection, but some of them ceased less than 1 month. Although smoking rate in the recent 1 month matched between MA abusers and HCs in the cross‐sectional study, it differed significantly in the longitudinal study. Previous studies suggested that smoking can lead to decreased diversity of intestinal microbiota,^82^ which can be reversible after cessation,^83^ but how long will this take is still not clear. Last but not least, this was an observational study, and the relationship between impulsivity and microbiota was solely numerical. Experimental studies are necessary to investigate the causational relationship between impulsivity and microbiota.

To summarize, the current study is the first to investigate the relationship between impulsivity and microbiota in individuals who abstained from MA abuse. Fecal microbiota dysbiosis was characterized by augmentation of opportunistic pathogens and reduction of probiotics, which was reversible after MA abstinence, highlighting the importance of abstention. Impulsivity was associated with characteristic dysbiosis induced by MA, and microbiota mediated the effect of MA abuse on impulsivity, indicating the important role of MGBA in the interrelationship between microbiota and behaviors and the potential of microbiota as a target for intervention of impulsivity.

## FUNDING INFORMATION

This work was supported by the National Key R&D Program of China (Grant #2018YFC1311600 and 2016YFC1306900 to Yanqing Tang), the Liaoning Revitalization Talents Program (Grant #XLYC1808036 to Yanqing Tang), and the Science and Technology Innovation 2030 – Major Project on Brain Science and Brain‐like Research (Grant #2021ZD0200600 and 2021ZD0200700 to Yanqing Tang).

## CONFLICT OF INTEREST STATEMENT

The authors declare no conflict of interest.

## Supporting information


Figure S1.
Figure S2.
Figure S3.

## Data Availability

The data that support the findings of this study are openly available in NIH National Library of Medicine at https://dataview.ncbi.nlm.nih.gov/object/PRJNA970410?reviewer=tqbb9rl8p3sei0k9ribqhkmnts, reference number PRJNA970410.
