# Supplementary material for: Associations between impulsivity and fecal microbiota in individuals abstaining from methamphetamine
Source: CNS Neurosci Ther. 2024 Feb 8;30(2):e14580. doi: 10.1111/cns.14580 (PMC10851322; doi:10.1111/cns.14580)
Supplement: Supplementary file 1 — Figure S1. Figure S2. Figure S3. [file CNS-30-e14580-s001.docx]

Supplementary


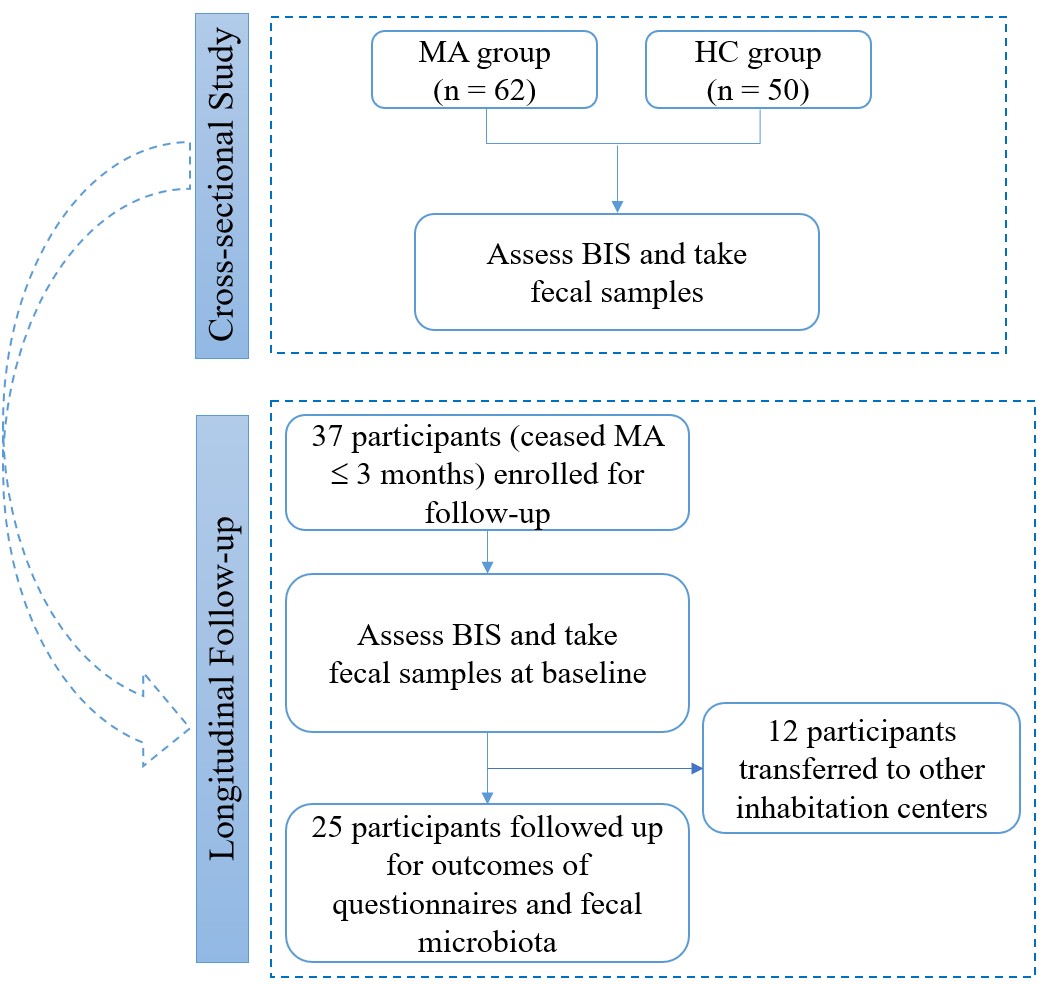


Fig. 1. Schematic overview of the procedure. In the cross-sectional study, BIS and fecal microbiota were compared between the MA group and HC group. Twenty-five participants who were absent from MA for less than 3 months were then followed up for 2 months, and BIS as well as the fecal microbiota were estimated at baseline and after a 2-month follow-up. Note: Barratt Impulsiveness Scale-11 (BIS).


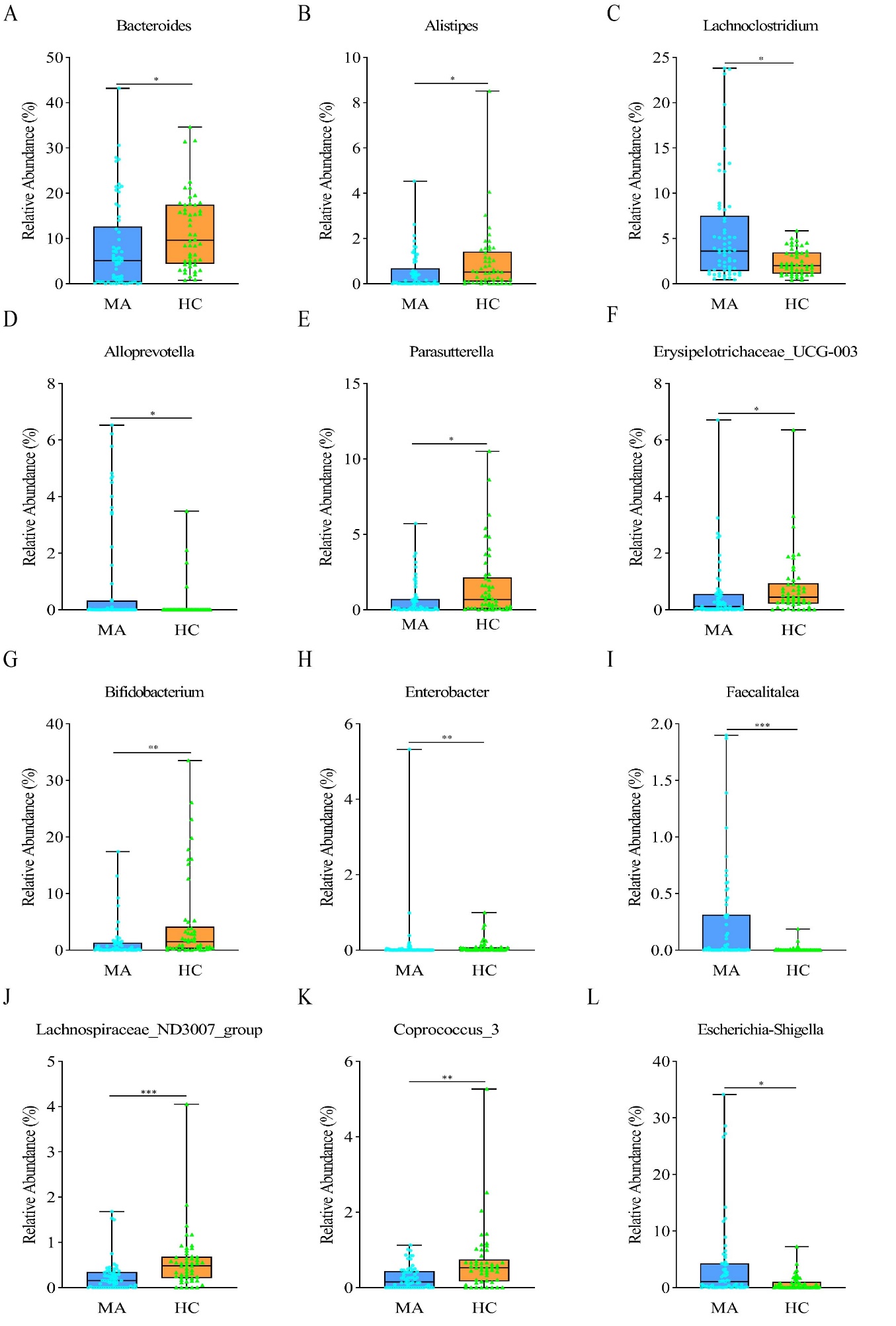


Fig. 2 Taxa with significantly different relative abundances between the MA and HC groups. The Wilcox test was computed for group-difference comparison, and FDR was applied for correction. The x-axis represents different groups, and the y-axis represents the relative abundance of the taxa. ***: q < 0.001, **: q < 0.01, *: q < 0.05.


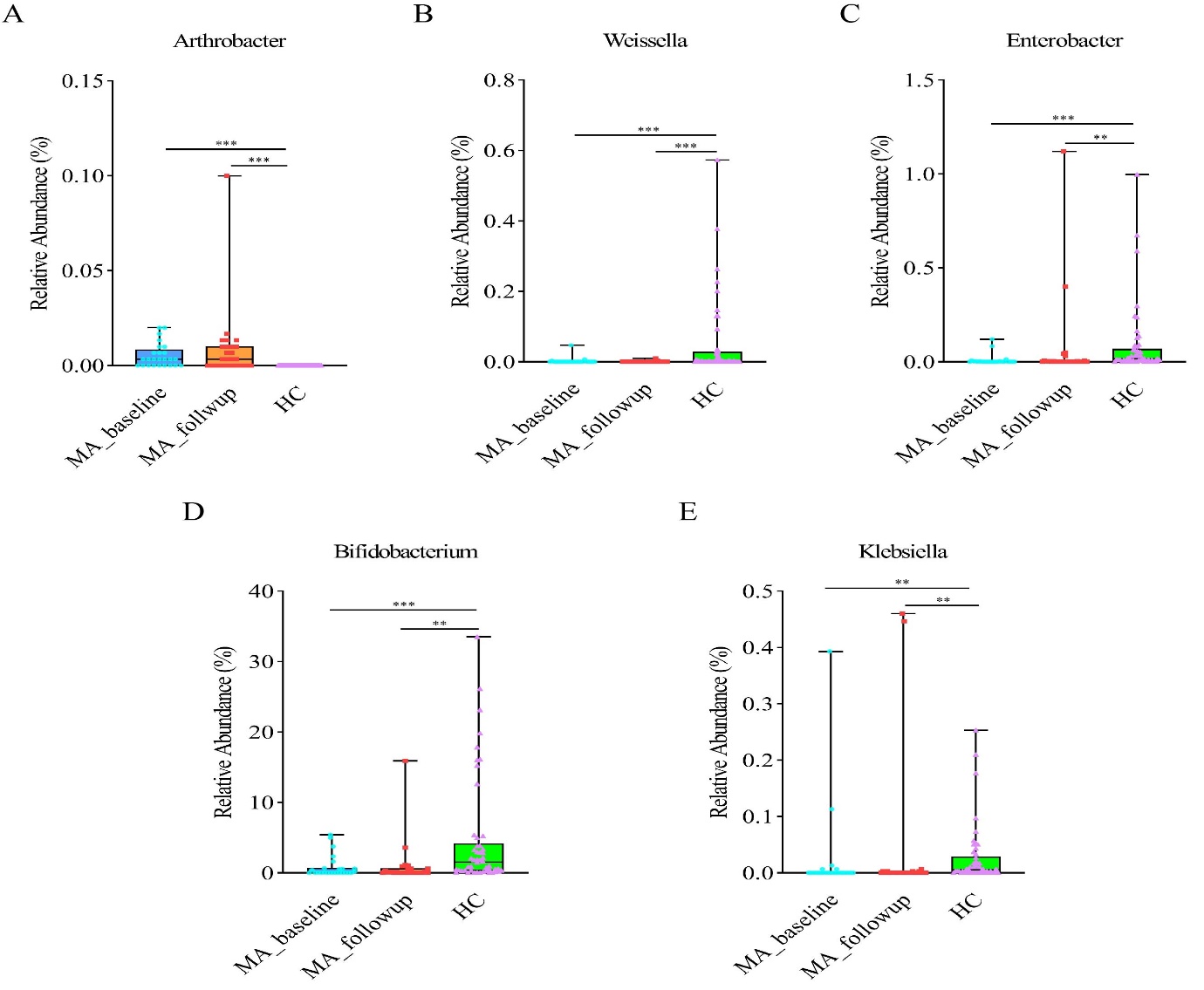


Fig. 3 Taxa with significantly different relative abundance across MA_baseline, MA_followup, and HC group. Kruskal-Wallis was calculated for group-difference comparison and FDR was applied for correction. The x axis represents different groups and the y axis was for the relative abundance of the taxa. ***: q < 0.001, **: q < 0.01, *: q < 0.05.
